# Association between parental decisions regarding abortion and severity of fetal heart disease

**DOI:** 10.1038/s41598-024-66027-8

**Published:** 2024-07-01

**Authors:** Masahiro Nakao, Masanari Kuwabara, Mika Saito, Chinami Horiuchi, Hiroko Morisaki, Kanako Kishiki, Yuji Hamamichi, Izumi Orui, Ryoko Ono, Ryo Suzuki, Miho Izawa, Yoshiki Maeda, Azumi Ohmori, Tomomi Uyeda, Satoshi Yazaki, Tadahiro Yoshikawa, Naoki Wada, Toru Hosoda, Masafumi Nii, Kayo Tanaka, Hiroaki Tanaka, Eiji Kondo, Yukihiro Takahashi, Tomoaki Ikeda

**Affiliations:** 1 Department of Obstetrics and Gynecology, Sakakibara Heart Institute, Tokyo, Japan; 2 Department of Obstetrics and Gynecology, Mie University Graduate School of Medicine, 2-174 Edobashi, Tsu, Mie 514-0001 Japan; 3 Division of Public Healh, Center for Community Medicine, and Division of Cardiovascular Medicine, Department of Medicine, Jichi Medical University, Shimotsuke, Tochigi Japan; 4 Department of Pediatric Cardiology, Sakakibara Heart Institute, Tokyo, Japan; 5 Department of Medical Genetics, Sakakibara Heart Institute, Tokyo, Japan; 6 Department of Pediatric Cardiac Surgery, Sakakibara Heart Institute, Tokyo, Japan; 7 Department of Cardiology, Sakakibara Heart Institute, Tokyo, Japan

**Keywords:** Congenital heart defects, Medical ethics, Risk factors, Outcomes research

## Abstract

The prenatal diagnosis of fetal heart disease potentially influences parental decision-making regarding pregnancy termination. Existing literature indicates that the severity, whether in complexity or lethality, significantly influences parental decisions concerning abortion. However, questions remain as to how fetal heart disease severity impacts parental decisions, given recent advancements in postsurgical outcomes. Therefore, we investigated risk factors associated with parents’ decision-making regarding abortion following a prenatal diagnosis of fetal heart disease. Our analysis included 73 (terminated: n = 37; continued: n = 36) pregnancies with a fetal heart disease diagnosed before 22 weeks of gestation. Increased gestational age at diagnosis reduced the likelihood of parents’ decision on termination (Model 1: adjusted odds ratio, 0.94; 95% confidence interval 0.89–0.99; Model 2: 0.95 0.90–0.997). Critical disease (5.25; 1.09–25.19) and concurrent extracardiac or genetic abnormalities (Model 1: 4.19, 1.21–14.53; Model 2: 5.47, 1.50–19.96) increased the likelihood of choosing abortion. Notably, complex disease did not significantly influence parental decisions (0.56; 0.14–2.20). These results suggest that parental decision-making regarding abortion may be influenced by earlier gestational age at diagnosis, the lethality of heart disease, and extracardiac or genetic abnormalities, but not its complexity if prenatal diagnosis and parental counseling are provided at a cardiovascular-specialized facility.

## Introduction

Recent advancements in perinatal management have notably improved the survival rates of infants diagnosed with fetal heart disease (FHD). Congenital heart defects (CHDs) are among the most prevalent forms of FHD, with a global birth prevalence increasing to 9–18 per 1000 live births^1,2^, remaining a significant cause of early infantile death and lifelong disability^2–5^. Additionally, while less frequent, other forms of FHD, such as cardiomyopathies and arrhythmias, are also observed. The establishment of antenatal screening for fetal structures has enabled prenatal detection and the implementation of preferred strategies for perinatal management^6–9^. However, recent studies suggest the potential implications of increased awareness and prenatal diagnosis of major CHD on parental decision-making regarding pregnancy^9,10^. It leads to a growing recognition of the importance of providing comprehensive support and counseling to expecting parents.

Several factors have been reported to influence parents’ decision-making regarding pregnancy following the diagnosis of FHD. Potential risk factors include severe heart diseases, extracardiac anomalies, genetic disorders, and early prenatal diagnosis^11–17^. Additionally, maternal age, level of education, marital status, and an extended period between suspicion and diagnosis can affect decision-making^18–21^. The impact of these factors may vary depending on cultural factors, healthcare systems, legal considerations, and related aspects. In Japan, termination of pregnancy (TOP) after 22 weeks of gestation, which is historically considered viable for the fetus, is strictly prohibited by law^22^. Additionally, governmental healthcare expense subsidy systems, covering the majority of CHD cases, help alleviate the burden of postnatal treatment for families^23^.

It is important to note that existing studies primarily defined severity based on structural complexity or potential lethality. However, the complexity of FHD may not consistently correlate with life-threatening implications for expectant mothers. Recent advancements in postsurgical outcomes have considerably increased the population of complex FHD cases reaching adulthood through multidisciplinary management^4,24^. Consequently, questions remain regarding how FHD severity influences parental decisions concerning TOP. Therefore, our study aimed to investigate the factors associated with parental decision on TOP in patients with FHD.

## Results

**Figure 1:**
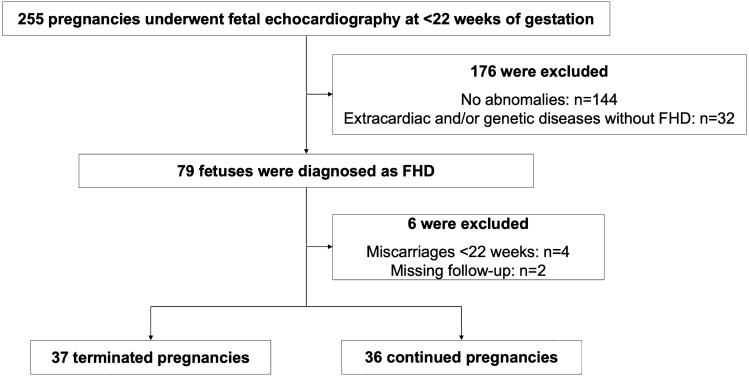
Flow diagram of the study groups. Among the 255 pregnancies that underwent fetal echocardiography at < 22 weeks of gestation, 79 fetuses were diagnosed with FHD. Of these, 73 cases were included in the final analyses. *FHD* fetal heart disease.

Among the 255 pregnancies that underwent fetal echocardiography before 22 weeks of gestation, 79 fetuses were diagnosed with cardiac disease. Excluding six cases due to miscarriages before parental decisions (n = 4) and missing follow-up (n = 2), a total of 73 fetuses (terminated: n = 37; continued: n = 36) were included in the analyses (Fig. 1). The clinical data on maternal background and prenatal diagnosis are outlined in Table 1. There were four unmarried individuals and one teenager, with no issues regarding parental acknowledgement. The interval between suspicion and the diagnosis was a median of three days (interquartile range (IQR) 1–7 days). Prenatal diagnoses conducted before 22 weeks of gestation identified 54 (74%) cases of complex FHD and 13 (18%) cases of critical FHD. Among these FHD cases, concurrent extracardiac or genetic abnormalities were noted in 26 (36%) cases, while 35 (48%) were affected by critical or concurrent extracardiac or genetic diseases. The extracardiac lesions were observed in craniofacial (n = 16), skeletal (n = 11), abdominal (n = 9), thoracic (n = 5), and genitourinary (n = 2) regions. Nineteen fetuses were strongly suspected of having a genetic disease based on ultrasound findings or genetic screening tests, with 10 of them confirmed through amniocentesis (trisomy 18: n = 9, trisomy 21: n = 6, 22q11.2 deletion: n = 2, trisomy 13: n = 1, and Turner syndrome: n = 1). The gestational age at diagnosis was earlier in fetuses with concurrent extracardiac or genetic abnormalities compared to those with isolated FHD (19.1 vs. 20.3 weeks, p < 0.01). However, no significant difference was observed in gestational age at diagnosis between complex and non-complex FHD (20.3 vs. 20.1 weeks, p = 0.42) or between critical and non-critical FHD (20.1 vs. 20.3 weeks, p = 0.27) (see Supplementary Table 1 online). The agreement between prenatal and postnatal diagnosis in the available cases (n = 27) was nearly perfect, with a Cohen’s kappa coefficient of 0.91. Nine out of 36 continued cases were excluded from the agreement analysis due to unavailable data on postnatal diagnosis (childbirth at the other hospital: n = 7, stillbirth: n = 2).

**Table 1:** Maternal and fetal characteristics.

	Terminated group (n = 37)	Continued group (n = 36)	p-value
Maternal characteristics			
Age (years)	34 (31–37)	34 (30–38)	0.93
Marital status			
Single	2 (5.4%)	2 (5.7%)	0.80
Married	35 (94.6%)	32 (91.4%)	
Re-married	0 (0%)	1 (2.9%)	
Nulliparous	20 (54.1%)	19 (52.8%)	0.91
In vitro fertilization	7 (18.9%)	4 (11.4%)	0.38
Prenatal diagnosis			
Gestational age at diagnosis (weeks)	19.3 (16.7–20.4)	21.0 (20.3–21.3)	< 0.01
Interval from suspicion to diagnosis (days)	4 (2–7)	3 (1–7)	0.86
Fetal sex (estimated)			
Female	15 (40.5%)	13 (36.1%)	0.91
Male	13 (35.1%)	13 (36.1%)	
Unclear	9 (24.3%)	10 (27.8%)	
Complex FHD	24 (64.9%)	30 (83.3%)	0.07
Critical FHD	10 (27.0%)	3 (8.3%)	0.04
Extracardiac/genetic abnormalities	21 (56.8%)	5 (13.9%)	< 0.01
Positive result of genetic screening test^a^	5/7 (71.4%)	1/4 (25.0%)	0.24
Positive result of amniocentesis	8/13 (61.5%)	2/7 (28.6%)	0.35

Data are presented as median (interquartile range) or number (percentage).

^a^Details of genetic screening test results (number of positive results/total tests; terminated vs. continued groups, respectively): non-invasive prenatal testing (NIPT; fetal DNA screening for trisomy 13, 18, and 21) (1/3 vs. 0/2); first-trimester ultrasound risk assessment interpreted with maternal age or combined exam with maternal serum marker test for trisomy 13, 18, and 21 (5/5 vs. 1/2); quad marker screening for trisomy 18 and 21, and neural tube defects (0/1 vs. 0/1). Please note that these values include duplicates.

*n* number, *y* years, *IQR* interquartile range, *FHD* fetal heart disease.

**Figure 2:**
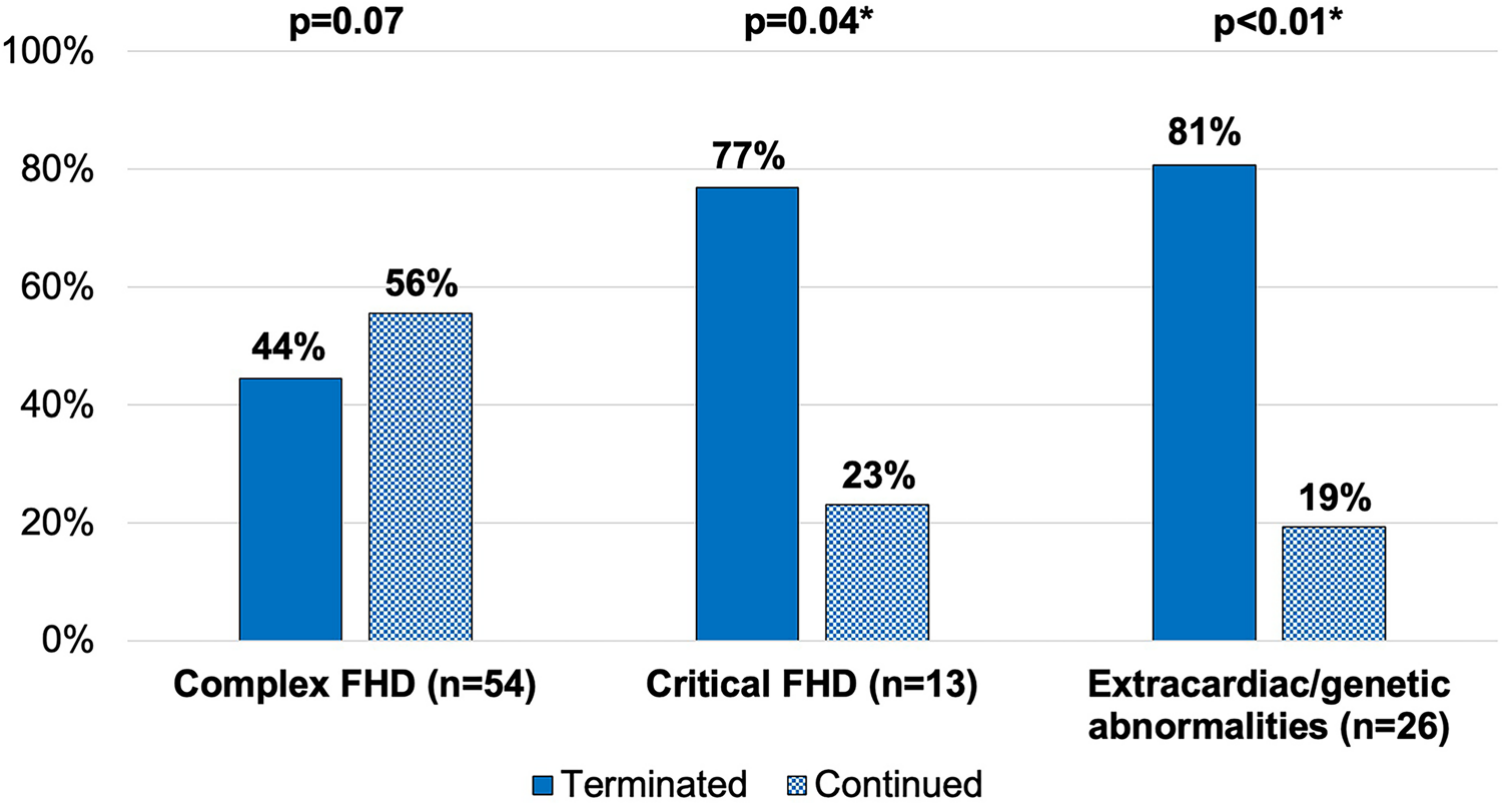
Prevalence of parental decisions regarding abortion in cases with complex and critical fetal heart diseases and those with concurrent extracardiac or genetic abnormalities. *Indicates statistical significance. *FHD* fetal heart disease.

In comparison to the cases in the continued group, fetuses in the terminated group received prenatal diagnosis earlier in gestation (19.3 weeks in the terminated group vs. 21.0 weeks in the continued group, p < 0.01), while the time intervals from suspicion did not significantly differ between the groups (4 days vs. 3 days, p = 0.86). Figure 2 illustrates the prevalence of parental decisions regarding abortion in cases with complex and critical FHD and those with concurrent extracardiac or genetic abnormalities. Nearly half of the complex cases (24 cases vs. 30 cases, p = 0.07) and approximately 80% of critical cases (10 cases vs. 3 cases, p = 0.04), as well as those with extracardiac or genetic abnormalities (21 cases vs 5 cases, p < 0.01), resulted in TOP. Multivariable logistic regression analyses showed that increasing gestational age at diagnosis was associated with a decreased likelihood of parents’ decision to terminate the pregnancy (Model 1: adjusted odds ratio (OR) 0.94, 95% confidence interval (CI) 0.89–0.99; Model 2: adjusted OR 0.95, 95% CI 0.90–0.997). Critical FHD (adjusted OR 5.25, 95% CI 1.09–25.19) and comorbid extracardiac or genetic abnormalities (Model 1: adjusted OR 4.19, 95% CI 1.21–14.53; Model 2: adjusted OR 5.47, 95% CI 1.50–19.96) were associated with an increased likelihood. However, complex FHD was not significantly associated with an increased likelihood (adjusted OR 0.56, 95% CI 0.14–2.20) (Table 2). In the individual diagnoses of FHD, 12 out of 13 fetuses with atrioventricular septal defect (AVSD), more than half of those with coarctation of the aorta (CoA) or interrupted aortic arch (IAA) (4 out of 7 cases combined), and tetralogy of Fallot (TOF, 5 out of 9) resulted in voluntary termination. Notably, among the aborted fetuses, 10 with AVSD, 3 with CoA or IAA, and 4 with TOF had a genetic disorder suspected through ultrasound or diagnosed via amniocentesis. Meanwhile, all six fetuses with transposition of the great arteries (TGA) were carried to live birth (see Supplementary Table 2 online).

**Table 2:** Multivariable logistic regression analysis for parental decisions on pregnancy termination.

Variables	Crude		Model 1		Model 2	
	OR	95% CI	OR	95% CI	OR	95% CI
Maternal age						
Per 1 year increased	1.02	0.92–1.12	1.01	0.90–1.14	1.01	0.90–1.14
Gestational age at diagnosis						
Per 1 day of gestational age increased	0.93	0.89–0.98*	0.94	0.89–0.99*	0.95	0.90–0.997*
Complex FHD						
Positive vs. negative	0.37	0.12–1.12	0.56	0.14–2.20	NA	NA
Critical FHD						
Positive vs. negative	4.07	1.02–16.31*	NA	NA	5.25	1.09–25.19*
Extracardiac/genetic abnormalities						
Positive vs. negative	8.14	2.58–25.62*	4.19	1.21–14.53*	5.47	1.50–19.96*

Model 1: Adjusted for maternal age, gestational age at diagnosis, complex heart disease, and extracardiac/genetic abnormalities.

Model 2: Adjusted for maternal age, gestational age at diagnosis, critical heart disease, and extracardiac/genetic abnormalities.

*Indicates significance at P < 0.05.

*OR* odds ratio, *CI* confidence interval, *FHD* fetal heart disease, *NA* not applicable.

## Discussion

This retrospective observational study conducted at a cardiovascular tertiary center in Japan indicates that critical FHD and comorbid extracardiac or genetic abnormalities were associated with an increased likelihood of parents' decisions to terminate pregnancies. Conversely, prenatal diagnosis of complex FHD may not influence parental decision-making. Additionally, increasing gestational age at prenatal diagnosis was associated with a decreased likelihood of termination.

Intriguingly, the complexity of FHD did not have a significant influence on parental decisions regarding TOP in this study. A recent study in a Taiwanese cohort showed that prenatal counseling by a pediatric cardiologist following the diagnosis significantly decreased the likelihood of choosing abortion^17^. Although we did not explicitly assess the impact of the facility level, our findings could be attributed to the presence of a specialized facility offering accurate diagnosis, detailed prognostic information, and comprehensive counseling services^25,26^. These factors assist couples in coping with fetal disease^21,27^, suggesting the importance of comprehensive support after prenatal diagnosis. We should also be aware of the considerable heterogeneity in the classification of “complex” based on the guidelines for adults with congenital heart disease (ACHD). Incorporating the possibility of single versus biventricular repair could clarify parental decisions regarding TOP. Conversely, in the cases deemed challenging, even at specialized facilities, the severity (i.e., lethality) potentially contributes to the decision-making process, emphasizing the critical need for detailed and transparent information about potential outcomes.

Consistent with the previous research, FHD cases accompanied by extracardiac or genetic abnormalities significantly influenced parents’ decisions regarding TOP in the present study. This finding is understandable, given that these patients face potential risks of surgical and neurodevelopmental challenges^28–31^. The high proportion of TOP following prenatal diagnosis of septal and conotruncal defects in our cohort may have been influenced by a well-known association with chromosomal disorders^32,33^. However, as mentioned earlier, the odds ratios might decrease if these cases were provided with more detailed prognostic information at specialized facilities covering extracardiac disorders.

Regarding gestational age at prenatal diagnosis, it is unclear whether earlier prenatal diagnosis directly enhances parental decisions regarding TOP. However, our findings suggest a potential association between the gestational age at diagnosis and the presence of concurrent extracardiac or genetic abnormalities, even after adjusting for these factors. Furthermore, the earlier diagnosis could be attributed to safer and more private options for TOP or premature prenatal attachment and reduced psychological distress that typically develops as pregnancy advances^20,34–36^. Alternatively, it might be due to the extended timeframe available for considering the option of abortion^37^, which may be attributed to the legal prohibition of abortion after 22 weeks in Japan. Therefore, this result may differ in other countries due to variations in cultural attitudes and abortion law.

This observation at a specialized facility indicates that parents’ primary concern, when considering TOP after prenatal diagnosis of fetal heart defect, is the potential lethality rather than its complexity. This underscores the essential role of clinical settings staffed by experienced professionals who can provide precise and comprehensive care to couples. When discussing options with expectant parents, healthcare providers should be aware of this concern. Nevertheless, it is essential to acknowledge the higher risk of adverse outcomes in cases with complex FHD compared to those with simple abnormalities, even in the current state of medical advancement^4^.

The present study highlights the impact of CHD severity on parental decision-making regarding TOP, suggesting that the primary concern for parents considering abortion is the potential lethality of the fetus or its lifelong complications rather than its complexity. This offers valuable insights for healthcare providers involved in prenatal diagnosis, counseling, and care, emphasizing the importance of continually providing comprehensive information to respective parents with consideration of these factors.

However, this study has several limitations, including potential selection bias due to the nature of retrospective observation and the specific facility setting. The study was conducted in a tertiary center specializing in cardiovascular diseases, where 74% of the cases involved complex FHD cases. Furthermore, nearly half (48%) were affected by critical or concurrent extracardiac or genetic diseases, significantly influencing the decision to terminate the pregnancy. This finding may explain the high proportion observed in the TOP group. Additionally, there are unmeasured, mainly socioeconomic factors unavailable from the current retrospective observation that can influence parental decision-making (e.g., maternal educational level, financial or social support availability, motivation behind the referral for detailed diagnosis). Further research comparing these factors within a comprehensive range of healthcare settings, including cardiac and non-cardiac disease cases, is warranted to enhance our understanding.

## Conclusion

In summary, this retrospective observational study conducted at a cardiovascular center in Japan indicates that an earlier gestation at prenatal diagnosis, critical FHD, and concurrent extracardiac or genetic abnormalities may influence parents’ decisions regarding TOP in patients with FHD but not in cases of complex FHD. These findings suggest that an environment offering comprehensive care by specialists after diagnosis may significantly contribute to successful support for parental decision-making. However, conclusions may differ in other countries due to variations in cultural attitudes and abortion law. Further research with a comprehensive range of healthcare settings, including cardiac and non-cardiac disease cases, is warranted for a better understanding.

## Methods

### Study population and data collection

A single-center, cross-sectional study was conducted at the Sakakibara Heart Institute, a tertiary center for cardiovascular diseases that offers prenatal diagnosis, prenatal and genetic counseling services, and perinatal care provided by experts. The study focused on pregnant women who underwent fetal echocardiography performed by experienced pediatric cardiologists and were diagnosed with FHD before 22 weeks of gestational age from May 2014 to March 2022. Participants who experienced a miscarriage before deciding on their pregnancy or for whom information about the decision was missing were excluded. Clinical data on maternal baseline information, gestational age at diagnosis, interval from suspicion to diagnosis, prenatal diagnosis before 22 weeks of gestation, parental decisions concerning their pregnancies (whether to continue or terminate), and postnatal diagnosis were collected through individual chart reviews. We assessed the factors associated with parents’ decisions regarding TOP. The accuracy of prenatal diagnosis was verified by comparing it with postnatal diagnosis made by pediatric cardiologists through ultrasonography after birth and computed tomography before surgery if applicable.

### Definitions

#### TOP

TOP was defined as parents’ voluntary decision to terminate their pregnancy before 22 weeks of gestation, which is the legal limit for abortion based on mutual agreement between the spouses^22^.

#### Severe (complex and critical) FHD

To assess the impact of FHD severity on the decision, we defined two types of “severe” diseases based on previous reports^11–16^, utilizing established classifications. Cases were categorized as “complex” FHD based on the ACHD anatomical and physiological classification, which is widely used in the ACHD guidelines (e.g., double outlet right ventricle, IAA, TGA, functionally single ventricle, or mitral atresia)^38–40^. Cases were considered “critical” if they involved high-risk heart diseases with a poor prognosis or anticipated an urgent requirement for immediate postnatal intensive care, aligning with either level 4 or 5 within the Level of Care Assignment and Coordinating Action Plan (e.g., hypoplastic left heart syndrome or TGA with restricted foramen ovale or an intact atrial septum, obstructed total anomalous pulmonary venous return, Ebstein anomaly with severe tricuspid regurgitation or hydrops, TOF with absent pulmonary valve, Arrhythmias with hydrops, or cardiomyopathy with ventricular dysfunction)^41^.

#### Extracardiac/genetic abnormalities

The FHD cases presenting concurrent extracardiac malformations or genetic diseases included those strongly suspected based on obstetric ultrasound findings or genetic screening tests or those confirmed via karyotyping tests.

### Statistical analysis

Continuous and integer variables are presented as median and IQR, while categorical variables are presented as numbers and percentages. The agreement between prenatal and postnatal diagnoses of FHD was analyzed using Cohen’s kappa coefficient. We performed univariate analyses between the two groups using the Wilcoxon signed-rank sum test for continuous variables and the chi-square or Fisher’s exact test for categorical variables. Crude ORs and 95% CIs were obtained using logistic regression models to assess the relationship between TOP and maternal age, gestational age at diagnosis, complex FHD, critical FHD, and extracardiac or genetic abnormalities. We examined the factors associated with parents’ decisions regarding TOP using multivariable logistic regression models. Covariates were applied based on potentially relevant factors to TOP reported in the peer-reviewed literature (as mentioned in the introduction section) and data availability within the chart review, including maternal age, gestational age at diagnosis, complex FHD or critical FHD, and extracardiac or genetic abnormalities. Notably, among these variables, the two categories of severe FHD—complex FHD, defined based on the structural criteria, and critical FHD, defined on the basis of anticipated prognosis or an urgent requirement for postnatal intensive care—often demonstrate overlap and are indicative of multicollinearity. Consequently, two logistic models were created. The first model was adjusted for maternal age, gestational age at diagnosis, complex FHD, and extracardiac or genetic abnormalities. In contrast, the second model was adjusted for maternal age, gestational age at diagnosis, critical FHD, and extracardiac or genetic abnormalities. The results of these regression analyses are presented as adjusted ORs along with 95% CIs. Statistical significance was considered at p < 0.05. All analyses were conducted using STATA version 17.0 (Stata Corporation, College Station, TX, USA).

### Ethical statement

The ethics committee of Sakakibara Heart Institute approved this study protocol (approved number 22-031, on August 29, 2022) and waived the requirement for informed consent due to the retrospective analysis of anonymized data. The research rigorously adhered to the Declaration of Helsinki, and this manuscript complies with the STROBE guidelines for reporting observational studies^42^.

### Supplementary Information

Supplementary Tables.

## Data Availability

Data are available on reasonable request to the corresponding author.
