# Supplementary material for: Association between parental decisions regarding abortion and severity of fetal heart disease
Source: Sci Rep. 2024 Jul 1;14:15055. doi: 10.1038/s41598-024-66027-8 (PMC11220094; doi:10.1038/s41598-024-66027-8)
Supplement: Supplementary file 1 — Supplementary Tables. [file 41598_2024_66027_MOESM1_ESM.pdf]

“**Association between parental decisions regarding abortion and severity of fetal heart disease**”

Masahiro Nakao, Masanari Kuwabara, Mika Saito, Chinami Horiuchi, Hiroko Morisaki, Kanako Kishiki, Yuji Hamamichi, Izumi Orui, Ryoko Ono, Ryo Suzuki, Miho Izawa, Yoshiki Maeda, Azumi Ohmori, Tomomi Uyeda, Satoshi Yazaki, Tadahiro Yoshikawa, Naoki Wada, Toru Hosoda, Masafumi Nii, Kayo Tanaka, Hiroaki Tanaka, Eiji Kondo, Yukihiro Takahashi, and Tomoaki Ikeda.

Supplementary Table 1. Relationship between gestational age at diagnosis and potential contributing factors

	Non-complex FHD	Complex FHD	p-value
Gestational age at diagnosis (weeks)	20.1 (14.7–21.3)	20.3 (19.3–21.0)	0.42

	Non-critical FHD	Critical FHD	p-value
Gestational age at diagnosis (weeks)	20.3 (19.0–21.1)	20.1 (18.1–20.6)	0.27

	Isolated FHD	Extracardiac/genetic abnormalities	p-value
Gestational age at diagnosis (weeks)	20.9 (19.9–21.3)	19.1 (13.6–20.3)	<0.01

Data are presented as median (interquartile range). Abbreviations: FHD, fetal heart disease.

Supplementary Table 2. Overview of prenatal diagnosis of fetal heart disease

Prenatal diagnosis	Terminated group (n=37)			Continued group (n=36)		
	Complex	Non-complex	Critical	Complex	Non-complex	Critical
Vascular ring (n=2)	0	0	0	1	1	0
Persistent left superior vena cava (n=2)	0	0	0	0	2	0
Ventricular septal defect (isolated, n=3)	0	1	0	0	2	0
Atrioventricular septal defect (n=13)	2	10	0	1	0	0
Coarctation of the aorta or interrupted aortic arch (n=7)	2	2	0	3	0	0
Ebstein anomaly or dysplastic tricuspid valve (n=2)	1	1	2	0	0	0
Tetralogy of Fallot (n=9)	5	0	2	4	0	0
Double-outlet right ventricle (n=12)	5	0	1	7	0	1
Pulmonary atresia (all forms, n=7)	2	0	1	5	0	0
Transposition of the great arteries (d-TGA or l-TGA, n=6)	0	0	0	6	0	0
Truncus Arteriosus (n=1)	1	0	1	0	0	0
Single ventricle [b] (n=26)	12	0	4	14	0	1
Total anomalous pulmonary venous return (n=5)	2	0	2	3	0	2
Heterotaxy (n=9)	3	0	1	6	0	2
Ventricular inversion (n=4)	2	0	0	2	0	0
Cardiomyopathy or myocarditis (n=1)	0	0	0	0	1	1
Cardiac tumour (n=1)	0	1	0	0	0	0
Arrhythmia (n=1)	0	1	0	0	0	0

The data contains duplicates because individual diagnoses of heart disease are listed separately. [b] Single ventricle includes tricuspid atresia, hypoplastic left heart, or any other anatomic abnormality with functionally single ventricle. Abbreviations: TGA, transposition of the great arteries.
